# Characteristics associated with quality of life in the early stages of Mycosis Fungoides

**DOI:** 10.22088/cjim.14.1.16

**Published:** 2023

**Authors:** Pedram Nourmohammadpour, Maryam Nasimi, Zeinab Aryanian, Azadeh Goodarzi, Reyhaneh Jahazi, Ifa Etesami

**Affiliations:** 1 Department of Dermatology, Razi Hospital, Tehran University of Medical Sciences, Tehran, Iran; 2 Autoimmune Bullous Diseases Research Center, Tehran University of Medical Sciences, Tehran, Iran; 3 Department of Dermatology, Babol University of Medical Sciences, Babol, Iran; 4 Department of Dermatology, Rasool Akram Medical Complex Clinical Research Development Center (RCRDC), School of Medicine، Iran University of Medical Sciences (IUMS), Tehran, Iran; 5 Department of Dermatology, Razi Hospital, Tehran University of Medical Sciences, Tehran, Iran

**Keywords:** Mycosis fungoides, Quality of life, Lymphoproliferative disorders

## Abstract

**Background:**

Mycosis fungoides (MF) is a lymphoproliferative disorder characterized by skin-homing atypical lymphocytes. This study aimed to evaluate the quality of life (QoL) in MF patients in the early stages of the disease and the associated factors using the Dermatology Life Quality Index (DLQI).

**Methods:**

Thirty MF patients (21 females/9 males) with a mean age of 46.73±15.9 years and in the early stages of the disease (26 stage Ι and 4 stage ΙΙ) were enrolled and were asked to fill the DLQI questionnaire.

**Results:**

The mean DLQI score was 9.93±5.89. The QoL was largely affected by the disease in near half of the patients (46.7% of patients had a DLQI score11-20). QoL was significantly correlated with educational level and was more impaired in patients with lower educational status (DLQI score spearman rho=-0.382, P=0.037). QoL was not associated with sex, age, disease stage and disease duration. The symptom and feeling dimension of DLQI was significantly more impaired in patients with both patch and plaque compared to patch only (spearman rho= 0.397, P= 0.03).

**Conclusion:**

This study demonstrates how largely patients’ QoL is influenced in the early stages of MF, especially in patients with lower educational levels.

Cutaneous T cell lymphoma (CTCL) refers to a group of heterogeneous lymphoproliferative disorder characterized by skin-homing atypical lymphocytes. The most common type of CTCL is mycosis fungoides (MF) with an annual incidence of 7.7 new cases per one million person-years. The median age of diagnosis is 55-60 and the disease affects men more commonly (1). Although MF has a chronic course, the majority of patients are in early stages with limited patches and plaques. Also, some patients advance to tumor stages and systemic involvement (2). The disease prognosis depends significantly on a stage with a range of 10-year survival of 98% for stage IA (limited patch or plaque) to 30-40% for stage IVA (3). Although various treatment modalities are available for MF including skin-directed therapies (psoralen, ultraviolet therapy, radiotherapy or total skin electron beam) and systemic therapies (retinoids, extracorporeal phototherapies, alpha interferon, chemotherapy and bone marrow transplantation), none of them is curative. In the previous studies by some researchers, it was demonstrated that MF has a significant impact on patients’ psychological status and quality of life (QoL), due to severe symptoms such as pruritus and pain (4, 5). The Dermatology Life Quality Index (DLQI) is a validated questionnaire for evaluating QoL in skin diseases and had been used to evaluate QoL in other skin cancers such as non-melanoma skin cancers (6). There has been little emphasis on the QOL of MF patients particularly with only early stages and its associated factors.

This study aimed to measure QoL in a population of MF patients in the early stages of the disease and the factors associated with poorer QoL.

## Methods

This observational study was approved by the Ethics Committee of Dermatology Department of Tehran University of Medical Sciences. Our data were collected from a tertiary health care hospital, Tehran, Iran. From March 2018 to March 2019, patients with MF disease referred to our phototherapy clinic for treatment, able to independently complete the DLQI questionnaire in English were enrolled in this study. The disease diagnosis was made by both clinical and histopathological features compatible with MF. The patients were asked to complete the DLQI questionnaire, verbal and written consents were taken from the participants after informing them about the study. The demographic data including age, gender, marital status, educational level, smoking and data of their disease characteristics including the site of skin lesions, stage of the disease, disease duration and patients’ current treatment modalities were obtained. DLQI introduced by Finlay and Khan is a self-explanatory questionnaire consisting of ten questions grouped into six categories including symptoms and feelings, daily activities, leisure, work and school, personal relationships and treatment (7). Each question is scored from 0 (not at all) to 3 (very) and the final DLQI score results from the sum of all questions ranging from 0 to 30. The higher the score, the more is QoL disturbed (0–1 = no effect, 2–5 = small effect, 6–10 =moderate effect, 11–20 = very large effect, 21–30 = extremely large effect). A Persian version of DLQI was made valid and reliable previously (8). 

In this study, the effect of disease characteristics and demographic data on the DLQI score was also evaluated.

**Statistical Analysis:** For data analysis, SPSS 16.0 was used. Continuous variables were reported as mean and standard deviation (SD). Categorical variables were reported as frequencies and percentages. Continuous variables in two groups were compared using independent sample t-test if normally distributed and if the distribution was skewed, non-parametric Mann-Whitney U test was applied. Pearson’s correlation coefficient was used for assessing the correlation between continuous variables. Association between QoL and demographic features of patients or symptoms of MF were evaluated by multiple regression.A two-tailed p< 0.05 was considered statistically significant.

## Results

Thirty MF patients were enrolled in this cross-sectional study, 21 (70%) were females and 9 (30 %) were males with a mean age of 46.73±15.9 years (range of 22-70). The demographic data are described in table 1. The mean DLQI score of patients was 9.93±5.89 (range 0-21). 

**Table 1:** Patients’ demographic data and disease characteristics

**Patients’ characteristics**	**Value**
Age (mean ± SD)	46.73±15.9
Sex Female Male	21 (70%) 9 (30%)
Marital status (%) Married Single	24 (80%) 6 (20%)
Educational level (%) Illiterate School University graduate	1 (3.3%) 10 (33.3%) 19 (63.4%)
Smoking Yes No	6 (20%) 24 (80%)
Lesion location Extremities Trunk and extremities Head and neck, trunk and extremities	3 (10%) 22 (73.3%) 5 (16.7%)
Lesion type Patch Patch and plaque Patch, plaque and tumor	16 (53.3%) 12 (40 %) 2 (6.7 %)
Lymph node involvement Yes No	3 (10%) 27(90%)
Disease duration Under 6 months 6 months- 1 year 1 year- 5 year More than 5 years	5 (16.7%) 7 (23.3%) 10 (33.3%) 8 (26.7%)
Treatment modalities Phototherapy Interferon and phototherapy Retinoid and phototherapy Retinoid, phototherapy and MTX Chemotherapy, radiotherapy and phototherapy	25 (83.3 %) 1 (3.3 %) 1 (3.3 %) 1 (3.3 %) 2 (6.7%)
MF stage I II	26 (86.7%) 4 (13.3%)

The quality of life was largely affected by MF in near half of the patients (46.7% of patients had DLQI scores of 11-20). The DLQI was not significantly different between men (mean DLQI= 10.86±6.35) and women (mean DLQI= 7.78±4.2) (P=0.22). Factors such as smoking, site of skin involvement, marital status, disease stage (I or II), type of skin lesion (patch only or with plaque or tumor) were not significantly associated with impaired QoL (table 2).

Although the mean total DLQI score was not significantly different between different groups of educational levels, we evaluated the correlation between total DLQI score and different dimensions of DLQI with patients’ educational level. We observed a significant correlation between educational level and DLQI score (spearman rho=-0.382, P=0.037), implicating that the higher the educational level, the less the DLQI score was and obviously the higher the QoL was shown.Interestingly, the personal relationships and treatment dimensions of DLQI were significantly correlated with educational level with spearman rho= -0.438 (P=0.015) and spearman rho=-0.538 (P=0.002), respectively. We did not observe any correlation between the extent of MF lesions (involving trunk only, trunk and extremities and trunk and extremities and head and neck), MF stage or type of MF lesions (patch only, patch and plaque) with DLQI (table 3). The symptom and feeling dimension of DLQI was significantly more impaired in patients with both patch and plaque compared to patch only (spearman rho= 0.397, P=0.03). Multiple regression of dummy variables for educational levels comparing university with school educational levels showed unstandard coefficients of B= -5.347 with standard error 2.15 and a p-value of 0.019. Also, multiple regression for association between treatment dimensions of DLQI with educational level showed an unstandard coefficient B: 0.87 with standard error 0.22 and p-value: 0.035.

The symptom and feeling dimension of DLQI was significantly more impaired in patients with both patch and plaque compared to patch only.

**Table2:** Association between DLQI dimension scores and patients’ characteristics

	**Symptom and feelings**	**Daily activities**	**Leisure**	**Personal relationships**	**Work and school**	**Treatment**	Total score*
Sex Female Male P-value	2.11 2.14 0.894	2.48 1.22 0.063	1.67 1.56 0.894	1.67 1.00 0.756	1.39 1.22 0.689	1.57 0.89 0.104	10.86 7.78 0.226
Marital status Married Single P-value	2.13 2.17 1.00	2.08 2.17 0.705	1.54 2.00 0.667	1.46 1.50 0.781	1.42 0.50 0.296	1.46 1.00 0.402	10.08 9.33 0.82
Smoking Yes No P-value	2.5 2.04 0.598	1.17 2.33 0.119	1.17 1.75 0.326	0.50 1.71 0.193	1.33 1.21 0.739	0.67 1.54 0.063	7.33 10.58 0.231

*Mann Whitney test

**Table 3:** Correlation between education level, MF stage, location of the lesions (Trunk, trunk and extremities, trunk and extremities and head & neck)

	**Symptom & feelings**	**Daily activities**	**Leisure**	**Personal relationships**	**Work & school**	**Treatment**	Total score*
Educational level Illiterate School University graduate	-0.263 0.160	-0.64 0.736	-0.168 0.374	-0.438 0.015*	-0.298 0.110	-0.538 0.002*	-0.382 0.037*
extent of MF lesions trunk only (1) 1 & extremities (2) (2) & head & neck	-0.082 0.665	-0.146 0.443	-0.155 0.414	-0.336 0.069	-0.045 0.814	-0.231 0.220	-0.264 0.159
Type of MF lesions Patch Patch & plaque	0.397 0.03*	-0.068 0.721	-0.092 0.629	0.051 0.787	0.032 0.867	0.089 0.638	0.069 0.718
MF stage Stage 1 Stage 2	0.34 0.066	0231 0.220	0.145 0.444	0.083 0.663	0.055 0.774	-0.135 0.476	0.205 0.278

*P<0.05 was considered significant.

**Table 4:** Comparison of patients with DLQI ranging from 0 to 10 and patients with DLQI ranging from 11 to 30

		**Patients with DLQI ranging from 0 to 10**	**Patients with DLQI ranging from 11 to 30**
Number		15	15
Mean age		46.73 (range 24 to 68) years	46.73 (range 22 to 70) years
Sex	Male	6 (40%)	3 (20%)
	Female	11 (60%)	12 (80%)
Marital status	Married	12 (80%)	12 (80%)
	Single	3 (20%)	3 (20%)
Educational level	Illiterate	0	1 (6.66%)
	School	3 (20%)	7 (46.66%)
	University graduate	12 (80%)	7 (46.66%)
Smoking	Yes	4 (26.66%)	2 (13.33%)
	No	11 (73.33%)	13 (86.66%)
Lesion Location	extremities	0	3 (20%)
	Trunk and extremities	11 (73.33%)	11 (73.33%)
	Head and neck, trunk and extremities	4 (26.66%)	1 (6.66%)
Lesion type	Patch	7 (46.66%)	9 (60%)
	Patch and plaque	7 (46.66%)	5 (33.33%)
	Patch, plaque and tumor	1 (6.66%)	1 (6.66%)
Lymph node involvement	Yes	1 (6.66%)	2 (13.33%)
	No	14 (93.33%)	13 (86.66%)
Disease duration	Under 6 months	3 (20%)	2 (13.33%)
	6 months- 1 year	3 (20%)	4 (26.66%)
	1 year- 5 year	4 (26.66%)	6 (40%)
	More than 5 years	5 (33.33%)	3 (20%)
Treatment modalities	Phototherapy	12 (80%)	13 (86.66%)
	Interferon and phototherapy	0	1 (6.66%)
	Retinoid and phototherapy	1 (6.66%)	0
	Retinoid, phototherapy and MTX	1 (6.66%)	0
	Chemotherapy, radiotherapy and phototherapy	1 (6.66%)	1 (6.66%)
MF stage	I	13 (86.66%)	13 (86.66%)
	II	2 (13.33%)	2 (13.33%)

## Discussion

Although QoL evaluation is an essential aspect of treatment assessment in cancer patients, it is not commonly applied in CTCL patients. DLQI and Skindex-29 are the two major QoL evaluating questionnaires in patients with dermatologic disorders. Determining QoL in MF patients may be a helpful tool for evaluating different treatment modalities. As in other cancers, such determinations help to identify variables with negative impacts on patients’ QoL (9). This study evaluated QoL in 30 MF patients in the early stages and also assessed the effect of patients’ demographic features and disease characteristics on their QoL using the DLQI score. The QoL was very largely impaired in near half of the patients (46.7 % of patients had a DLQI score between 11 and 20) with a mean DLQI of 9.93±5.89. QoL was significantly correlated with educational level, which was more impaired in patients with lower educational status especially in the personal relationship and treatment dimensions (total DLQI score spearman rho=-0.382, P= 0.037). Moreover, the symptom and feeling dimensions of DLQI were significantly more impaired in patients with both patch and plaque lesions compared to patch only (spearman rho= 0.397, P=0.03), though the total DLQI score was not significantly correlated with the stage of disease or type of lesions.

In a recent study of 238 newly diagnosed MF or sezary syndrome patients using the Skindex-29 questionnaire, QoL was significantly worse in female patients, Sezary syndrome, advanced stages, alopecia and erythema (4). In our study, QoL was not significantly different between male or female patients. Some studies failed to find a gender difference in illness perception and QoL in CTCL patients similar to our study (10). In a study by Semenov et al. on 67 CTCL patients using Ontario Health Utilities Index Mark III (HUI3) questionnaire, a validated health utility instrument that evaluates the patients’ general health status and QoL and allows measuring quality-adjusted life-year (QALY), CTCL patients were shown to have significantly impaired quality of life in terms of an average loss of 1.47 QALY per patients (11). In addition, they showed that compared to other chronic diseases such as hemodialysis, diabetes mellitus and all-cancer patient population, QoL is more impaired in CTCL patients (11). A qualitative research held by Beynon et al. interviewing 19 CTCL patients, revealed that the disease and its physical symptoms significantly impacted the patient’s QoL and their relationships (12). Another study on one hundred CTCL patients using Skindex-29 and visual analog scale for itch (VASitch) found that all QoL aspects were impaired by disease, more advanced disease stage and more severe pruritus were correlated with more disturbed QOL (5). Unlike the previous study, we could not find any association between disease stage and QoL. One explanation can be the absence of advanced stages in our study and only early stages were enrolled in this study. Therefore, the effect of disease severity could not be detected. 

Studies evaluating QoL in the early stages of MF are so limited. Interestingly, a recent study using Skindex-29 in the early stages of MF has revealed that health-related QoL was impaired in the early stages of MF regardless of having a clinically evident disease or not without any significant difference (13). However, in our study, patients with both patch and plaques had a poorer status in symptom and feeling dimension of DLQI compared to patch only patients, implicating the impairing effect of disease progression on patients’ QoL. While comparing the effect of MF disease on patients’ QOL with other dermatological issues, it is important to note that MF disease even in early stages with a mean DLQI score of 9.93±5.89 in our study has as a quite large effect on QOL as pemphigus Vulgaris with a mean DLQI score of 10.9±6.9 (14). Furthermore, MF disease in early stages disturbs QOL more than an obviously QOL impairing disease such as alopecia areata with a mean pooled DLQI score of 6.3 (15). In contrast to our findings, in another study evaluating quality of life in 30 patients with stage I of MF, it was found that patients with early-stage MF have low QoL-impairment (16). Notably, larger studies show that the QOL in MF patients is not significantly affected in early stages of the disease and patients with advanced stages are more likely to manifest an impaired QOL; This fact has been approved in recent studies meaning that patients with more advanced MF (>IIA) have a worse QoL overall (4, 17, 18). In this regard, our study did not include a great number of patients with advanced stages of the disease which makes it a limitation to our study.

In our study, we did not find any correlation between the extent of MF lesions and QOL. In this vein, other studies indicate that QoL is affected more by disease stage than by body area or extent (19). To the best of our knowledge, most of the previous studies on QoL in MF patients were performed on patients with advanced MF stages or sezary syndrome. In this study, only patients with early MF stages (stage Ι or ΙΙ) were included, therefore, the adverse effect of advanced stages was not evaluated properly by this study.Notably, one of the limitations of this study is the small sample size and larger studies with greater sample size are recommended. Another limitation is the significant difference in number of participants in each group which causes impairment in comparison. Of note, this study showed that MF patients’ QoL even in the early stages is largely affected by the disease. Mucocutaneous diseases or systemic diseases that have dermatological symptoms can significantly affect the quality of life of patients, owing to they have visible symptoms and are often considered a cosmetic problem, therefore, many studies are performed on their effect on other aspects of patients’ life and on the new treatments all over the world (20-26) and neoplastic skin diseases are no exception to this rule and perhaps their effect on the mental dimensions of patients is logically more important and widespread. 

The QoL was largely impaired in nearly half of MF patients in the early stages of the disease, QoL was poorer in patients with lower educational level and the dimension of symptoms and feeling of DLQI score was more disturbed in patients with both patch and plaque lesions compared to patch only. Few studies have evaluated the quality of life in the early stages of MF and most studies indicate poorer QOL in MF patients with advanced stages. Hence, less attention has been paid on the QOL of patients with early stages of MF. This study demonstrates how QoL is influenced by MF disease even in early stages and how essential it is to educate patients with lower educational levels on their disease aspects who are more prone to worse QoL.
